# Remote Ischemic Preconditioning (RIPC) Modifies Plasma Proteome in Humans

**DOI:** 10.1371/journal.pone.0048284

**Published:** 2012-11-05

**Authors:** Michele Hepponstall, Vera Ignjatovic, Steve Binos, Paul Monagle, Bryn Jones, Michael H. H. Cheung, Yves d’Udekem, Igor E. Konstantinov

**Affiliations:** 1 Haematology Research, Murdoch Childrens Research Institute; Melbourne, Victoria, Australia; 2 Cardiac Surgery Unit and Cardiology, Royal Children’s Hospital; Melbourne, Victoria, Australia; 3 Department of Paediatrics, The University of Melbourne; Melbourne, Victoria, Australia; 4 Bioscience Research Division, Department of Primary Industries, Melbourne, Victoria, Australia; University of Cincinnati, United States of America

## Abstract

Remote Ischemic Preconditioning (RIPC) induced by brief episodes of ischemia of the limb protects against multi-organ damage by ischemia-reperfusion (IR). Although it has been demonstrated that RIPC affects gene expression, the proteomic response to RIPC has not been determined. This study aimed to examine RIPC induced changes in the plasma proteome. Five healthy adult volunteers had 4 cycles of 5 min ischemia alternating with 5 min reperfusion of the forearm. Blood samples were taken from the ipsilateral arm prior to first ischaemia, immediately after each episode of ischemia as well as, at 15 min and 24 h after the last episode of ischemia. Plasma samples from five individuals were analysed using two complementary techniques. Individual samples were analysed using 2Dimensional Difference in gel electrophoresis (2D DIGE) and mass spectrometry (MS). Pooled samples for each of the time-points underwent trypsin digestion and peptides generated were analysed in triplicate using Liquid Chromatography and MS (LC-MS). Six proteins changed in response to RIPC using 2D DIGE analysis, while 48 proteins were found to be differentially regulated using LC-MS. The proteins of interest were involved in acute phase response signalling, and physiological molecular and cellular functions. The RIPC stimulus modifies the plasma protein content in blood taken from the ischemic arm in a cumulative fashion and evokes a proteomic response in peripheral blood.

## Introduction

Ischemic preconditioning is a potent innate mechanism observed in many species whereby cells develop tolerance to ischemia-reperfusion (IR) injury when exposed to controlled periods of transient, sub-lethal ischemia prior to a prolonged ischaemia [1], [2] **.** However, *local* ischemic preconditioning is not clinically applicable to most patients. During the past decade, a simple technique of preconditioning has been developed with the potential for rapid translation into clinical practice [3].

Remote ischemic preconditioning (RIPC) is a phenomenon where brief episodes of ischemia of one tissue (e.g., skeletal muscle) protect against IR injury in an organ at a *remote* location [4]. RIPC has great potential for clinical application as it can be applied non-invasively using a standard blood pressure cuff to induce cycles of IR to skeletal muscle [3], [5]. We have previously demonstrated that brief episodes of limb ischemia protected the donor heart after transplantation [6], providing multi-organ protection against cardiopulmonary bypass-induced tissue injury [7] and effective protection during evolving myocardial infarction [8]. We have also demonstrated that IR and RIPC induced a genomic response in the myocardium and circulating leukocytes of experimental animals and in humans [9]–[11]. Additionally, we observed that RIPC decreased expression of kinin receptors [12], neutrophil adhesion and also modified the functional responses of human neutrophils [13]. We have also applied RIPC to clinical practice and demonstrated, in a randomized controlled trial, the benefits of the RIPC in children undergoing cardiac surgery [14]. A recent large randomized controlled trial further demonstrated a beneficial effect of the RIPC, as a complement to angioplasty, on myocardial salvage in patients with acute myocardial infarction [5].

Although the clinical benefits of RIPC are apparent, the mechanism underlying this protection remains unknown. Others and we have previously suggested the existence of a blood-borne effector of the RIPC stimulus that is transferred from the transiently ischemic limb to remote organs rendering them resistant to prolonged ischemia [6], [15]. Furthermore, it appears that transient limb ischemia not only remotely preconditions through a humoral mechanism, but also that plasma transfer from the ischemic limb of one species may protect against IR injury in other species [15].

It is intuitive to believe that the observed changes in gene expression in response to the RIPC [9], [10] will result in changes protein expression. However, the proteomic response to RIPC has not been studied to date. The purpose of this study was to determine 1) if the plasma from the transiently ischemic limb has a modified proteomic profile, 2) if the proteomic changes are cumulative with each subsequent episode of transient ischemia, and 3) if the RIPC stimulus evokes a global proteomic response early and late after the induction of the transient limb ischemia.

## Materials and Methods

**Figure 1:**
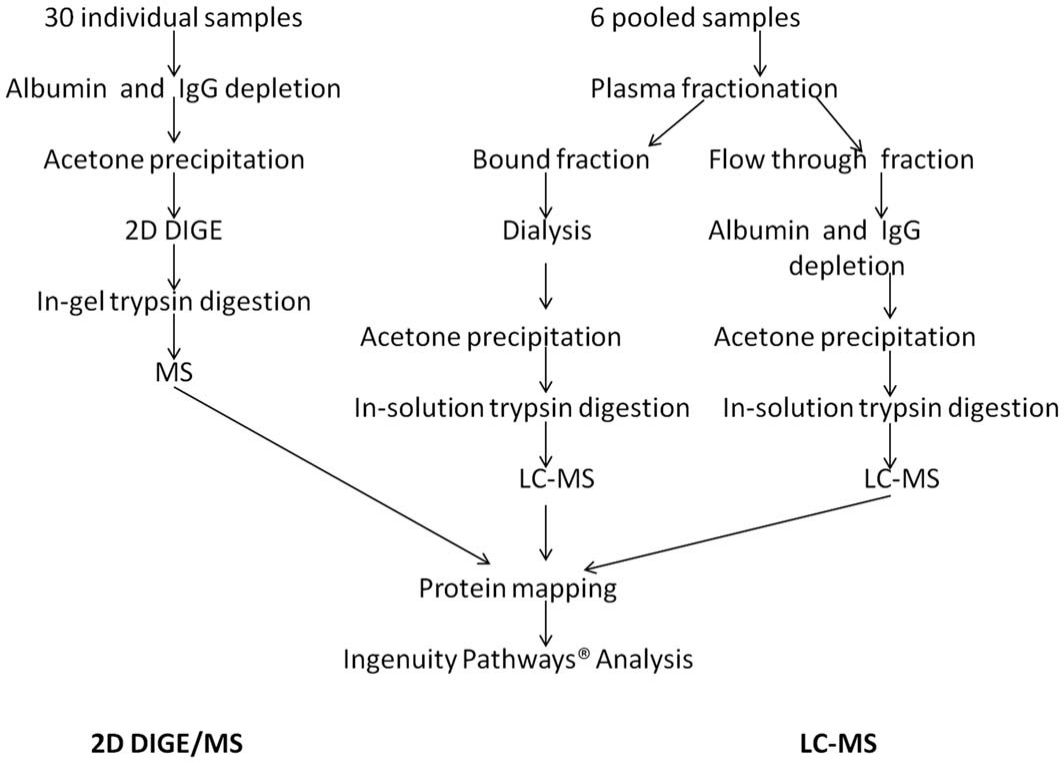
Two complementary proteomic methods used to assess RIPC induced changes in the plasma proteome. 2D DIGE- 2Dimensional Difference in gel electrophoresis, LC– Liquid chromatography, -MS-mass spectrometry.

This study was approved by the Royal Children’s Hospital Ethics in Human Research Committee (#29007) and written informed consent was obtained from the participants. Five healthy adult male volunteers 36.2±6.3 (mean ± SD), not on any medications were fasted overnight and underwent the RIPC protocol. The protocol consisted of 4 cycles of 5 minutes of ischemia alternating with 5 minutes of reperfusion. Ischemia was induced by inflating a standard blood pressure cuff to a pressure exceeding systolic, as previously described [14]. Venous blood samples were collected from the same arm at 6 time-points: baseline, at the beginning of each period of re-perfusion and then at 15 minutes and 24 hours following application of the RIPC stimulus. Blood samples were collected in S-Monovette® tubes (Sarstedt, Australia), containing 1 volume of citrate per 9 volumes of blood. The samples were centrifuged at 3000 rpm for 10 min at 10°C (Megafuge 1.0R, Heraeus), the plasma was collected and stored at −80°C. The samples were then analysed using two methods described below (**Figure 1**).

### Two-Dimensional Difference in Gel Electrophoresis (2D-DIGE) and Mass Spectrometry

The analysis was conducted on 30 individual samples (6 samples from 5 individuals).

Albumin and IgG depletion was performed using the albumin IgG depletion kit (GE Healthcare, Australia). The remaining proteins were precipitated using acetone precipitation, as specified in the depletion kit and resuspended in buffer containing 7 mol/L urea, 2 mol/L thiourea, 4% 3-[3-cholaamidopropyl]-1-propane-sulfonate and 30 mmol/L Tris. The protein content of each sample was quantified using the Bradford assay (Bio-Rad, Hercules, CA, USA) and bovine serum albumin standards [16].

**Table 1:** Significantly changed proteins 2D DIGE/MS.

Accession number	Protein	Protein score^□^	p-value(t-test)	Average ratio	Main function
gi 178751	α2-antiplasmin precursor	64	0.046	1.37	Serine protease inhibitor
gi 4557321	Apolipoprotein A-1*	197	0.045	−1.21	Lipid transport
gi 8101268	Complement C3	393	0.0047	1.46	Immune response
gi 223002	Fibrin beta*	294	0.028	1.18	Haemostasis
gi 223170	Fibrinogen gamma*	198	0.018	1.24	Haemostasis
gi 8853069	Vitronectin precursor	120	0.014	−1.32	Cell adhesion

* Proteins that were also found to change significantly using LC-MS.

□The protein score indicates the confidence with which the proteins identified match the NCBInr human protein database. Only scores greater than 40 were considered to match with sufficient confidence. Average ratio indicates the degree of difference in the abundance between two protein spot groups. Values below zero indicate a down-regulation, whereas, values greater than zero indicate up-regulation.

**Figure 2:**
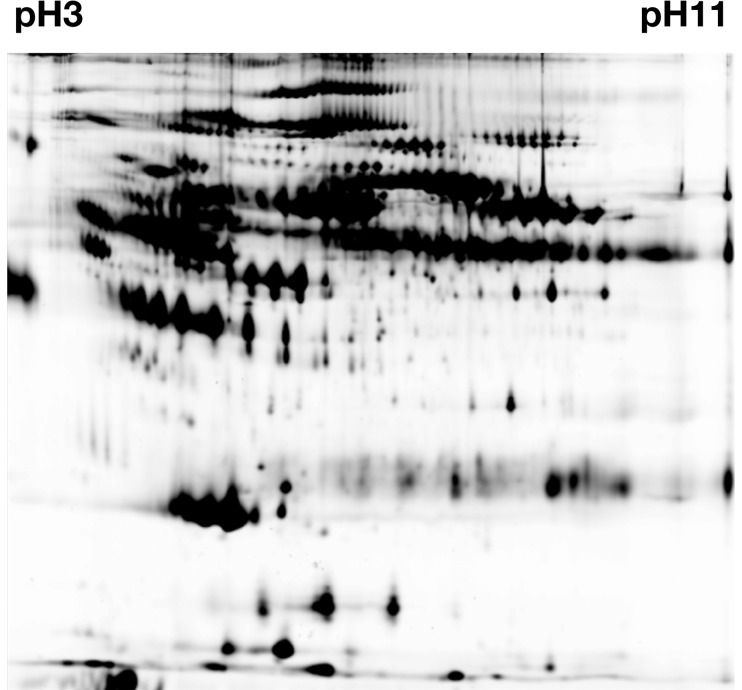
The protein pattern from a representative 2D-DIGE gel of human plasma proteins. pH 3 to pH 11 - left to right.

The internal standard, consisting of an equal amount of each of the 30 samples, was labelled with Cyanine 2 (Cy2) fluorescent dye (GE Healthcare, Australia) and run on each gel to control for gel-to-gel variation. Each sample was randomised to be labelled with either Cy 3 or Cy5 dye and then randomized to 15 gels. The Cy2, Cy3 and Cy5 samples (50 mg of sample/400 pmol of Cy dye) for each gel were pooled and loaded onto the Immobilized pH Gradient (IPG) strip. One 24 cm, pH 3–11 strip per gel was rehydrated with 15 ml IPG buffer and 3 ml DeStreak solution (GE Healthcare, Australia). Proteins were separated based on isoelectric point (first dimension) and molecular weight (second dimension) using previously published methodology [16]. Gels were scanned using the Typhoon Trio variable mode imager (GE Healthcare, Australia) [16]. Data obtained from the scanning were quantified using DeCyder software version 6.5 (GE Healthcare, Australia). The Differential In-gel analysis (DIA) was used to optimize spot detection. The Biological Variation Analysis (BVA) module was used for analysis of each sample according to the corresponding time point. The filtering parameters were set to determine protein spots that had a p-value of <0.05 and a 1.5-fold change in abundance between the time points.

**Table 2:** Significantly changed proteins using LC-MS.

Protein	Cluster number*	Accession number	Protein	Protein score	Main function
**1**	03064; 03114	gi152207506	Alpha-1-antitrypsin	50.52	Major plasma protease inhibitor
**2**	0355; 01053; 01172; 01516; 01772; 02364; 02525; 02607; 02613; 02623; 02632; 03082; 03101; 03169; 03213	gi3212456	Albumin	1409.65	Maintenance of osmotic pressure (carrier protein)
**3**	03271; 03316; 03379	gi4502027	Albumin pre-proprotein	3915.51	Albumin synthesis
**4**	0851	gi4502067	Alpha-1-microglobulin/Bikunin precursor	760.52	Trypsin inhibitor
**5**	02542; 02625	gi2098275	Amyloidogenic TransthyretinVariants	717.28	Molecular Transport
**6**	1075	gi999513	Antithrombin Iii ComplexChain A,	1280.26	Protease inhibition
**7**	00345; 00421; 00991; 01738; 02127; 02426; 03248; 03288; 03368	gi90108664	Apolipoprotein A-I	221.85	Lipid Transport
**8**	01759; 00319; 01798; 02803; 03173	gi24987503	Apolipoprotein A-Ii	605.36	Lipid Transport
**9**	03249	gi619383	Apolipoprotein D	476.73	Lipid Transport
**10**	0822	gi6573461	Apolipoprotein H	1950.32	Lipid Transport
**11**	03243	gi4262120	Beta-globin	119.95	Haemoglobin synthesis
**12**	0393	gi218511956	Complement C1r	591.36	Immune response
**13**	1014; 0920	gi81175238; gi1314244	Complement C4B	2498.95	Immune response
**14**	0868	gi21730336	Complement C8 gamma	564.78	Immune response
**15**	1056; 0797; 0524; 0480; 0412; 0230; 0102; 0681	gi119625338	Fibrin beta	321.18	Haemostasis
**16**	0781; 0400	gi223170	Fibrinogen gamma	395.17	Haemostasis
**17**	1015; 0927; 0853	gi109658664	Fibronectin 1	2796.95	Endothelial cell activation
**18**	1072; 0237	gi4504165	Gelsolin precursor	1365.75	Actin binding
**19**	01892; 02104	gi169791771	Haemoglobin	470.28	Oxygen binding
**20**	01942	gi63080988	Haemoglobin alpha-2globin mutant	470.28	Oxygen binding
**21**	00935	gi47679339	Haemoglobin beta	110.75	Oxygen binding
**22**	03201; 02348; 02396	gi4826762; gi229323; gi296653	Haptoglobin	653.78	Haemoglobin binding
**23**	02059	gi45580723	Haptoglobin 2-alpha	400.55	Acute phase response
**24**	01237	gi119589124	Hemopexin, isoform	504.33	Heme binding
**25**	0900; 0793	gi4504489	Histidine-rich glycoproteinprecursor	1076.52	Protein binding
**26**	01381	gi229536	Immunoglobulin A Light chain	439.93	Acute phase response
**27**	02489; 03175	gi8569502	Immunoglobulin G-1 (Fc Fragment)	1139.00	Acute phase response
**28**	01081; 01369	gi184747	Immunoglobulin G-1 heavy chain constant region	353.79	Acute phase response
**29**	02730	gi25987833	Immunoglobulin G-2 heavy chain constant region	427.39	Acute phase response
**30**	02372	gi311771988	Immunoglobulin G-Aptamer Complex	570.83	Acute phase response
**31**	0653; 00247	gi2414492	Immunoglobulin heavy chainconstant region	275.38	Acute phase response
**32**	0937	gi553485	Immunoglobulin kappa chainvariable region	117.71	Acute phase response
**33**	0777	gi3328006	Immunoglobulin light chainvariable region	92.82	Acute phase response
**34**	1049; 0875; 0770; 03057	gi166007160	Immunoglobulin M	840.10	Acute phase response
**35**	0884; 0779; 0218	gi4467842	Immunoglobulin M heavy chain	105.89	Acute phase response
**36**	0942; 0925; 0421; 0356; 0344	gi55958063	Inter-alpha (globulin) inhibitor H2	1816.20	Protease inhibition
**37**	1024; 0892; 0871; 0801; 0762; 0662	gi225311	Lipoprotein B100	5942.27	Lipid transport
**38**	0374	gi156616294	N-acetylmuramoyl-L-alanineamidase precursor	705.96	Peptidoglycan biosynthesis
**39**	0465	gi160877748	Neuropilin-1 B1 Domain In Complex With A Vegf-Blocking Fab, Chain L	909.96	Protein signalling
**40**	0994; 0889; 0632; 0348; 0168	gi8569387	P14-Fluorescein-N135q-S380c-Antithrombin-Iii, Chain I	1280.26	Protease inhibition
**41**	0519	gi229528	Protein Len, Bence-Jones	687.73	Immune response
**42**	03436	gi229526	Protein Rei, Bence-Jones	558.14	Immune response
**43**	01545	gi223069	Protein Tro alpha 1 H	707.61	Immune response
**44**	00132; 00479; 01819; 00088; 00270; 00395; 00603; 00910; 00978; 01032; 01972; 01602; 01127; 01975; 02244; 03107; 3362	gi110590597; gi194383506; gi110590599	Transferrin	3130.44	Iron binding
**45**	01415	gi1881852	Sry-related HMG box gene	101.3	DNA binding
**46**	02051; 02692	gi18655424	Vitamin D Binding Protein	994.05	Vitamin D sterol transport
**47**	03398	gi139641	Vitamin D-binding protein precursor	1023.55	Vitamin D sterol transport
**48**	02516	gi4699583	Zinc-Alpha-2-Glycoprotein	145.06	Lipid transport

Cluster number refers to the number alocated to each peptide fragment in the Genedata software. Accession number refers to the corresponding protein from the NCBInr human protein database. Protein score is a score assigned by the Proteome Discoverer software to indicate the confidence with which the proteins identified match the NCBInr human protein database. Only Protein scores greater than 40 were considered to match with sufficient confidence.

* - Each cluster number equals a unique piptide identified for the particular protein.

Proteins of interest were excised from the gels robotically using the Ettan Spot-picker (GE Healthcare, Australia) and prepared for in-gel trypsinolysis as previously described [16]. Gel plugs were consecutively washed with 25 mM NH_3_HCO_3_ followed by 50% v/v acetonitrile for 15 min each. Following dehydration by incubation at 37°C for 30 min, gel plugs were incubated with modified porcine trypsin in 25 mM NH_3_HCO_3_ (Promega) (pH 9, 37°C, 15 h). Trifluoroacetic acid (0.5% w/v) was subsequently added to neutralise the trypsin. The digested proteins were concentrated directly onto a thin layer affinity matrix solution of α-cyano-4-hydroxycinnamic acid for analysis by MALDI-TOF MS. The MS reflector mode was used to generate a protein mass fingerprint for the identification of each protein (4700 Proteomics Analyzer, Applied Biosystems, USA), operating at a resolution of 10,000–15,000 FWHM (Full Width at Half Maximum). Reordered in positive reflector mode at a laser intensity of 2950, spectra were acquired at 200 Hz using a YAG laser (335 nm). A mass filter that excluded matrix cluster ions and trypsin autolysis peaks was applied. Ten of the most intense peptide ions were selected for further MS analysis (MS/MS). All MS/MS data from the TOF-TOF was acquired using a default 1 kV method at laser energy 3000–3500. The PMF and MS/MS data were combined and submitted for database searching as described in the protein mapping section below. Protein identity was listed for samples that gave a significant (P<0.05). A peptide mass tolerance of 100 ppm and up to 1 missed cleavage allowed when searching against all databases.

**Figure 3:**
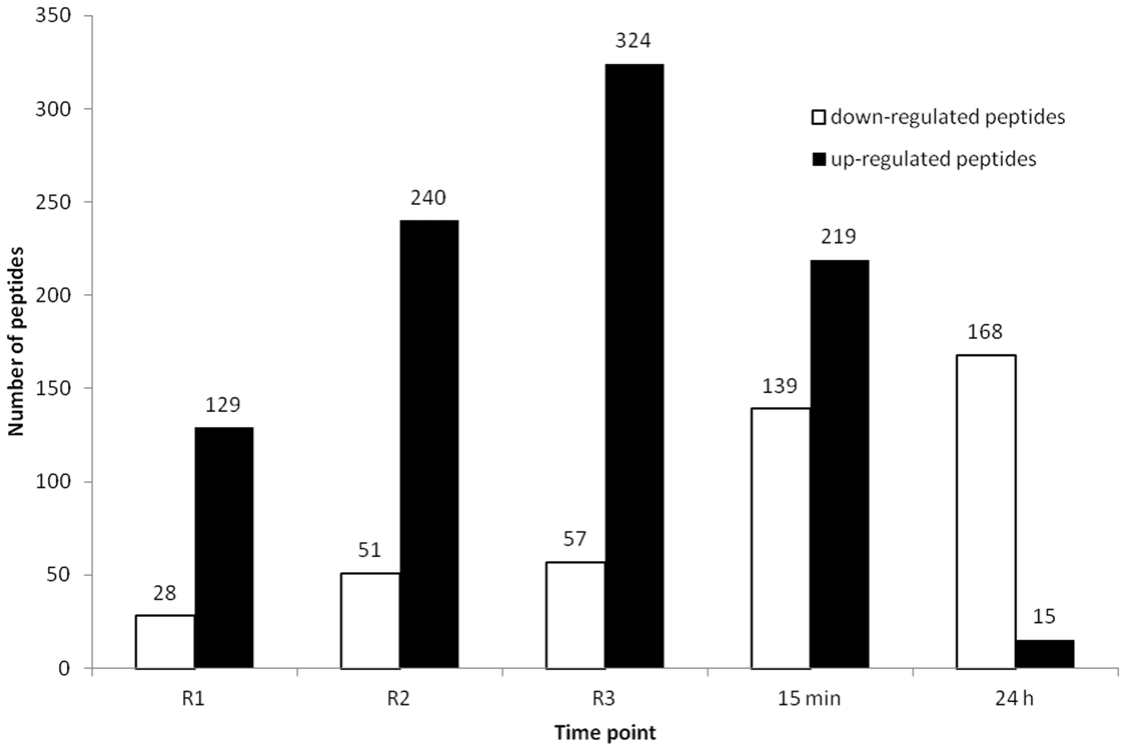
The number of peptides that changed significantly compared to the baseline. R1, R2 and R3 refer to the blood samples taken immediately after the first, second and third period of transient limb ischemia catching the blood coming from the ischemic arm at the beginning of each cycle of reperfusion (R), demonstrating a cumulative proteomic response to the RIPC stimulus. The remaining two samples were taken from the same arm at 15 minutes and 24 hours after completion of the RIPC stimulus, demonstrating an early and late global proteomic response to the RIPC stimulus.

### Liquid Chromotography (LC) and Mass Spectrometry

This method was applied to better assess the heparin-bound proteins. The analysis was conducted on 6 pooled samples from 5 individuals taken at 6 time points. Plasma fractionation was performed using the ÄKTA™ Fast Protein Liquid Chromatography (Amersham Pharmacia Biotech AB, Uppsala, Sweden). The plasma proteins were separated into two fractions, based on their affinity to heparin [17]. One fraction contained proteins that bind heparin (*bound fraction*) and the other fraction contained those that do not bind heparin (*flow through fraction*) [17]. Plasma samples were diluted 1∶3 in 50 mMTris-HCl, 0.1 M NaCl pH 7.5 (Kjellberg 2006) and passed through a 0.22 µM spin filter (Agilent Technologies, Australia) by centrifugation for 5 min. The samples were then fractionated in duplicate runs by injecting 400 µL of the sample into the AKTA system, through a 1 mL Hi-Trap Heparin column (GE healthcare, Australia) at a flow rate of 1 ml/min for 5 mins to collect the flow through fraction. The bound fraction was then eluted off the column under high salt conditions with 50 mM Tris-HCl, 3.0 M NaCl pH 7.5 for 13 mins (Kiellberg 2006). Between each sample run, the column was re-equilibrated with 50 mMTris-HCl, 0.1 M NaCl pH 7.5 for 7 minutes. Samples from the *bound* fraction were dialysed against phosphate buffered saline to reduce the salt concentration in preparation for acetone precipitation. Dialysis was performed for 48 hours with a change of buffer at 24 hours, with 25 mm×16 mm cellulose dialysis tubing (Sigma Aldrich, St Louis, USA) [18].

**Table 3:** Differentially expressed proteins coming from the ischemic arm demonstrating up regulation and down regulation.

Proteins up regulated	Proteins down regulated
α2-antiplasmin precursor	Alpha-1-microglobulin/Bikunin precursor
Albumin	Antithrombin Iii Complex, Chain A
Albumin pre-proprotein	Apolipoprotein H
Alpha-1-antitrypsin	Complement C1r
Amyloidogenic Transthyretin Variants	Complement C4B
Apolipoprotein A-I	Complement C8 Gamma
Apolipoprotein A-Ii	Gelsolin precursor
Apolipoprotein D	Histidine-rich glycoprotein precursor
Beta-globin	Immunoglobulin heavy chain constant region
Fibrin beta	Immunoglobulin light chain variable region
Fibronectin 1	Immunoglobulin M
Haemoglobin	Immunoglobulin M heavy chain
Haemoglobin alpha-2 globin mutant	N-acetylmuramoyl-L-alanine amidase precursor
Haemoglobin beta	Neuropilin-1 B1 Domain In Complex With A Vegf-Blocking Fab, Chain L
Hemopexin, isoform	P14-Fluorescein-N135q-S380c-Antithrombin-Iii Chain I,
Haptoglobin	Protein Len, Bence-Jones
Haptoglobin 2-alpha	Vitronectin precursor
Immunoglobulin A Light chain	
Immunoglobulin G-Aptamer Complex	
Immunoglobulin G-1 (Fc Fragment)	
Immunoglobulin G-1 heavy chain constant region	
Immunoglobulin G-2 heavy chain constant region	
Immunoglobulin kappa chain variable region	
Inter-alpha (globulin) inhibitor H2	
Lipoprotein B100	
Protein Rei, Bence-Jones	
Protein Tro alpha 1 H	
Sry-related HMG box gene	
Transferrin	
Vitamin D Binding Protein	
Vitamin D-binding protein precursor	
Zinc-Alpha-2-Glycoprotein	

**Figure 4:**
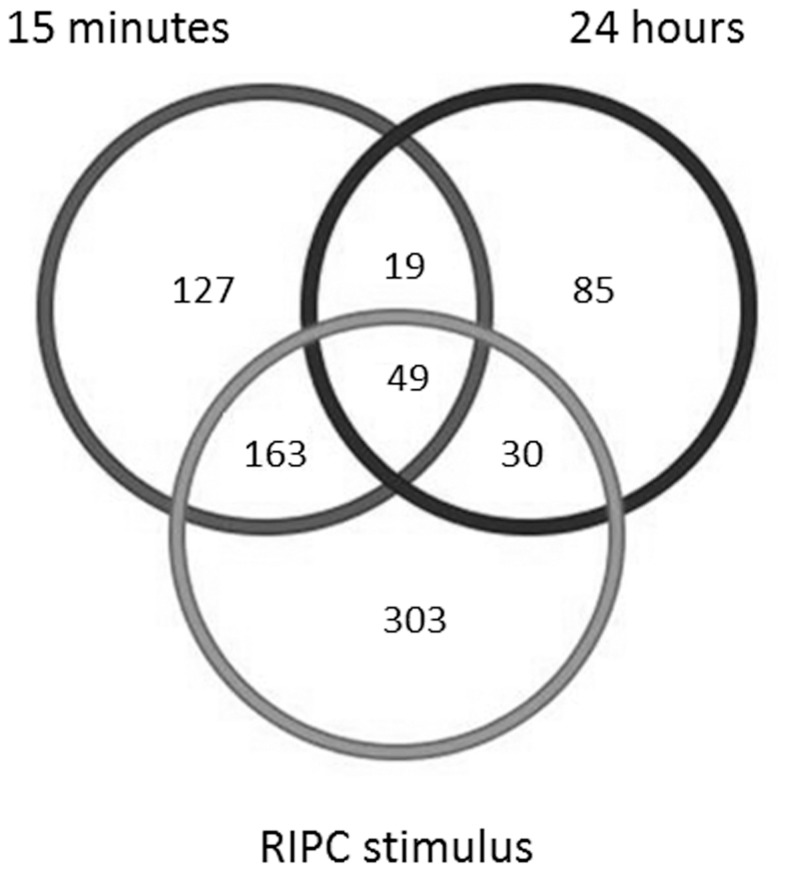
Venn diagram demonstrating the number of peptides that changed significantly during transient limb ischemia (combining all R1, R2, and R3 reperfusion periods), as well as at 15 minutes and 24 hours after the RIPC stimulus, compared with the baseline sample.

**Table 4:** Differentially expressed proteins in response to the RIPC stimulus demonstrating up regulation and down regulation during the early response (15 min).

Proteins up regulated	Proteins down regulated
Albumin	Alpha-1-microglobulin/Bikunin precursor
Albumin pre-proprotein	Antithrombin Iii Complex, Chain A
Alpha-1-antitrypsin	Apolipoprotein H
Amyloidogenic Transthyretin Variants	Complement C4B
Apolipoprotein A-I	Complement C8
Apolipoprotein A-Ii	Complement C1r
Apolipoprotein D	Gelsolin precursor
Beta-globin	Histidine-rich glycoprotein precursor
Complement C3	Immunoglobulin heavy chain constant region
Fibrinogen gamma	Immunoglobulin light chainvariable region
Fibrin beta	Immunoglobulin kappa chain variable region
Fibronectin 1	Immunoglobulin M
Haemoglobin	Immunoglobulin M heavy chain
Haemoglobin alpha-2 globin mutant	Lipoprotein B100
Hemopexin, isoform	N-acetylmuramoyl-L-alanine amidase precursor
Haptoglobin	Neuropilin-1 B1 Domain In Complex With A Vegf-Blocking Fab, Chain L
Haptoglobin 2-alpha	P14-Fluorescein-N135q-S380c-Antithrombin-Iii, Chain I
Haemoglobin beta	Protein Len, Bence-Jones
Immunoglobulin A Light chain	
Immunoglobulin G-Aptamer Complex	
Immunoglobulin G-1 (Fc Fragment)	
Immunoglobulin G-1 heavy chain constant region	
Immunoglobulin G-2 heavy chain constant region	
Inter-alpha (globulin) inhibitor H2	
Protein Rei, Bence-Jones	
Protein Tro alpha 1 H	
Sry-related HMG box gene	
Transferrin	
Vitamin D Binding Protein	
Vitamin D-binding protein precursor	
Zinc-Alpha-2-Glycoprotein	

**Table 5:** Differentially expressed proteins in response to the RIPC stimulus demonstrating up regulation and down regulation during the late response (24 h).

Proteins up regulated	Proteins down regulated
Amyloidogenic Transthyretin Variants	Albumin
Apolipoprotein A-Ii	Albumin pre-proprotein
Apolipoprotein D	Alpha-1-antitrypsin
Fibrinogen gamma	Alpha-1-microglobulin/Bikunin precursor
Fibrin beta	Antithrombin-Iii Complex, Chain A,
Hemopexin, isoform CRA_a	Apolipoprotein H
Haptoglobin 2-alpha	Apolipoprotein A1
Immunoglobulin A Light chain	Complement C4B
Protein Rei, Bence-Jones	Complement C8
Protein Tro alpha 1 H	Complement C1r
Sry-related HMG box gene	Fibronectin 1
Transferrin	Gelsolin precursor
Zinc-Alpha-2-Glycoprotein	Histidine-rich glycoprotein precurso
	Haemoglobin
	Immunoglobulin heavy chain constant region
	Immunoglobulin light chain variable region
	Immunoglobulin G-1 (Fc Fragment)
	Immunoglobulin G-1 heavy chain constant region
	Immunoglobulin G-2 heavy chain constant region
	Immunoglobulin kappa chain variable region
	Immunoglobulin M
	Immunoglobulin M heavy chain
	Inter-alpha (globulin) inhibitor H2
	Lipoprotein B100
	N-acetylmuramoyl-L-alanine amidase precursor
	Neuropilin-1 B1 Domain In Complex With A Vegf-Blocking Fab, Chain L,
	P14-Fluorescein-N135q-S380c-Antithrombin-Iii, Chain I
	Protein Len, Bence-Jones
	Vitamin D Binding Protein
	Vitamin D-binding protein precursor

Albumin and IgG were depleted from the *flow through fraction* using the Albumin and IgG removal kit (GE Healthcare, Australia). This was performed to increase the probability of detecting low abundance proteins that are not bound to heparin. The *bound fraction* was not subjected to this depletion protocol as albumin and IgG do not bind to heparin and are therefore not present in this fraction.

Both the *bound fraction* and the *flow through fraction* underwent acetone precipitation and quantification [16]. In-solution trypsin digestion was performed and samples prepared for MS using a standard protocol where four volumes of ice cold acetone were added to the samples and precipitation was carried out overnight at −20°C. Protein pellets were obtained by centrifugation at 13 000 g for 20 mins at 8°C and were resuspended in 6M Urea, 100 mM Tris buffer. The protein content of each sample was quantified using the Bradford assay (Bio-Rad, Hercules, CA, USA) by comparing against a standard curve of bovine serum albumin concentration [19]. In-solution trypsin digestion was performed on 50 µg of protein from each sample. The samples were reduced with 10 mM dithiothreitol for one hour, followed by alkylation with 55 mM iodoacetamide for one hour. The concentration of urea was reduced to <1M by diluting the sample with 0.4M Tris buffer at pH 7.8. Sequencing grade porcine trypsin (Promega, Madison, WI, USA) was added at a ratio of 1∶20 and trypsin digestion then carried out overnight at 37°C. The reaction was stopped by titration with concentrated acetic acid until the pH was lower than pH 6.

Following trypsin digestion, samples were passed through Oasis HLB extraction cartridges (Waters, Ireland) preconditioned with methanol and equilibrated with 2% acetonitrile and 0.1% Trifluroacetic acid (TFA). Bound peptides were first eluted with 80% acetonitrile containing 0.1% TFA, followed by 100% acetonitrile and 0.1% TFA. The combined eluant was lyophilised by freeze drying, after which each was reconstituted in 200 µL of 0.1% formic acid in preparation for mass spectrometry.

LC MS/MS was carried out on a LTQ Orbitrap Velos (Thermo Scientific, West Palm Beach, FL, USA) equipped with a nanoelectrospray interface coupled to an Ultimate 3000 RSLC nanosystem (Dionex, Sunnyvale, CA, USA). The nanoLC system used an Acclaim Pepmap nano-trap column (Dionex – C18, 100 Å, 75 µm×2 cm) and an Acclaim Pepmap RSLC analytical column (Dionex - C18, 100 Å, 75 µ m×15 cm). Typically for each LCMSMS experiment 1 µl of each peptide preparation, equating to 250 ng total peptide, was loaded onto the enrichment (trap) column followed by separation and elution of peptides from the analytical column employing a gradient from 3% to 45% acetonitrile over 90 minutes. The LTQ-Orbitrap Velos mass spectrometer was operated in the data dependent mode with nano ESI spray voltage of +1.6 kv, capillary temperature of 250°C and S-lens RF value of 60%. All spectra were acquired in positive mode with full scan MS spectra scanning from m/z 300–2000 in the flight time mode at 60,000 resolution after accumulating to a target value of 1.00e6 with maximum accumulation of 500 ms. The 8 most intense peptide ions with charge states ≥2 were isolated at a minimum threshold value of 2000 and fragmented by low energy collision induced dissociation (CID) with normalized collision energy of 35, activation Q of 0.25 and activation time of 10 ms. A dynamic exclusion of 1 repeat over 10 sec with exclusion duration of 15 sec was set. At all times, monoisotopic precursor selection was enabled. Each sample was run in triplicate with 2 blank injections between each triplicate set to minimize the effect of sample carryover [20]. Data processing was carried out using Expressionist Refiner MS (Genedata, Basel, Switzerland) to align MS data, carry out noise reduction, and for peak extraction (clustering). Clustering of MS/MS spectra was employed to identify the spectra of the same peptide (from triplicate runs) and to replace them with a single representative spectrum. Once clustered, peak area intensity measurements of precursor ions were extracted and analysed for statistical relevance using Genedata Analyst to compare each of the 3 samples collected post ischemia and 15 minutes and 24 hours thereafter with the baseline sample. Subsequently, peptides showing up or down-regulated expression (p<0.001 above a computer generated false discovery rate) across the time-points were collated into lists for identification using a targeted MS/MS approach. For biomarker discovery using targeted mass spectrometry analysis, the mass spectrometer was operated in the data-dependant mode as described above with the following modifications. The 10 most intense peptide ions with charge states ≥2 were isolated at a minimum threshold value of 2000 from an assigned parent list. A dynamic exclusion of 4 repeats over 30 sec with exclusion duration of 15 sec was set.

The MS data was loaded onto Proteome Discoverer 1.2 software suite (Thermo Scientific, West Palm Beach, FL, USA) and submitted to Mascot v.2.2.04 (Matrix Science, London, UK) www.matrixscience.com) to match against the National Centre for Biotechnology Information (NCBInr), Bethesda, US database. An initial filter of precursor mass was set between 300 to 6000 Da. The peptide mass tolerance was set to 20 ppm and 0.8 Da for MS/MS fragmentation ions Searches were carried out on the latest version of the NCBInr human database (National Centre for Biotechnology Information, Bethesda, US). Enzyme specificity was trypsin with a maximum of 2 missed cleavages. Cysteine carbaidomethylation (+57.0215 Da) and methionine oxidation (+15.9949 Da) were set as the fixed and variable modification respectively for all searches. ESI-FTICR was set as the default instrument search setting. All the spectra were searched against the decoy database to achieve a targeted false discovery rate of 1%. Only those peptides that matched the database with medium (FDR <0.05) or high confidence (FDR <0.01), ie protein score greater than 40, with spectra that matched the original data analysis for fragmentation pattern, retention time and mass to charge ratio were considered when assigning a positive match. Individual MS/MS spectra from the targeted runs within a precursor tolerance of 2 ppm and maximum R/T difference of 1.5minutes were merged (clustered) into single representative spectrum.

## Results

Using the 2D DIGE (**Figure 2**) with individual plasma samples, 33 spots were determined to have changed significantly in response to RIPC, p<0.05. From these protein spots, 6 proteins were successfully identified by MS and are presented in **Table 1**.

Using LC-MS analysis, 806 peptides were differentially expressed compared with the baseline sample (p<0.001), and of these, 133 (16.5%) peptides were successfully mapped to 48 known proteins in the NCBInr database (**Table 2**). The remaining peptides could not be matched to proteins currently available in the database.

The number of up-regulated peptides increased with reperfusion (**Figure 3**), and the number of up-regulated peptides peaking at 324 peptides following the third cycle of ischemia. Similarly, the number of down-regulated peptides increased steadily throughout the RIPC protocol, with the highest number of down-regulated peptides observed 24 hours following application of the RIPC stimulus.

The number of peptides that changed significantly compared to the baseline sample during the RIPC protocol as well as at 15 minutes and 24 hours after the RIPC stimulus is presented in **Figure 4**. Proteins that were differencially expressed at the various timepoints are shown in **Table 3** **,** **4** **and** **5**.

Three of the proteins were identified using both experimental approaches. These proteins were fibrin beta, fibrinogen gamma and apolipoprotein A. The main pathway involved in the RIPC response was acute phase response signalling.

## Discussion

A multi-organ protection by RIPC can be transferred to the target organs via humoral factors in plasma [6], [15]. Those factors may or may not be proteins. However, proteomic changes in plasma are an important component of the inflammatory response to IR injury. Thus, it appeared logical to examine proteomic changes in plasma induced by transient limb ischemia.

To the best of our knowledge, this is the first study to examine the global proteomic changes in plasma induced by brief forearm ischemia. Arrell et al., demonstrated in a rabbit model that pharmacologically induced preconditioning evoked proteomic changes in the myocardium [21]. Although proteomic changes in the target organ are of great interest, we focused on describing proteomic changes in plasma, in light of evidence that transfer of plasma from the transiently ischemic limb induced RIPC in the target organs [15].

Proteomic evaluation of plasma is challenging due to the high abundance proteins that obscure lower abundance proteins [22], [23]. We depleted two most abundant proteins – albumin and IgG. Although we attempted to deplete the samples of these high abundance proteins, residual albumin and IgG were still present. Since neither albumin nor IgG binds to heparin, further separation by fractionation based on the ability to bind to heparin was helpful. Thus, we used two different, but complementary methodological proteomic approaches in order to better define the proteomic changes in plasma. Fractionation and LC permitted a better evaluation of the heparin bound fraction and effectively cleared the albumin and IgG to further unmask lower abundance proteins. By analysing both fractions, we ensured that the majority of the plasma proteome was assessed.

The results of this study demonstrated that plasma proteome changes occurred during the RIPC and were cumulative with each episode of IR. The number of peptides in plasma coming from the ischemic arm increased with each episode of transient arm ischemia. These peptides were predominantly up-regulated (**Figure 3**). In contrast, at 15 minutes and 24 hours after the RIPC stimulus the peptides were predominantly down-regulated. The latter finding is consistent with our previous genomic study that demonstrated predominant down-regulation of pro-inflammatory gene expression early and late after the RIPC stimulus [9].

We identified 51 proteins which were differentially expressed in response to the RIPC protocol compared to baseline when the results of the two approaches were combined. The proteins identified, play a role in a range of cellular functions including immune response, haemostasis, haemoglobin binding and synthesis, protease inhibition, acute phase response, iron binding, lipid transport, oxygen binding, heme binding, vitamin D transport, protein binding, maintenance of osmotic pressure, trypsin inhibition, molecular transport and protein signalling, endothelial cell activation, actin binding, peptidoglycan biosynthesis and DNA binding. This suggests that the mechanisms involved in RIPC may involve a complex interaction of multiple redundant pathways such that there is regulation of cells surviving or yielding to ischemic damage. Many proteins identified in our study are biomarkers of cardiovascular disease [24].

Alpha-1-antitrypsin is one such protein that has been shown to contribute to protection of the kidney in a mouse model of IR injury through the initiation of the acute phase response to injury and exerting anti-apoptotic and anti-inflammatory effects [25]. We found this protein to be up regulated during the RIPC protocol as well as 15 minutes later consistent with its known role as an initiator of the acute phase response during injury. Haptoglobin is another acute phase protein with apparent involvement in IR injury. The level of haptoglobin is decreased during IR injury and normalized by preconditioning, attenuating the IR injury [26]. We found consistently higher levels of haptoglobin at all time points analysed compared to baseline.

Apolipoproteins were shown to be predominantly up-regulated during the RIPC stimulus as well as during the early and late after it. Apolipoproteins prevent endothelial dysfunction and inhibit lipid oxidation in models of myocardial and renal IR injury [27], [28] and may play a role in protection against IR injury. In particular, apolipoprotein A1 is involved in IR injury and has anti-inflammatory activity [28]. Apolipoprotein A1 protected against IR injury through suppression of intercellular adhesion molecule-1 and p-selectin expression, thus, decreasing neutrophil adhesion and subsequent tissue injury that resulted in improved cardiac contractility. It also reduced release the of creatnine kinase, tumor necrosis factor-alpha and other inflammatory cytokines and myeloperoxidase serum levels post ischemic insult [28]–[30]. Arrhythmias (ventricular tachycardia and ventricular fibrillation) associated with IR can be attenuated by lipoproteins [31].

Two complement proteins (C1r and C8) were down-regulated in our study during and after the RIPC stimulus. This is consistent with previous studies that demonstrated the gene expression of these proteins to be down regulated in the myocardium of rabbits in vivo [32] and in isolated rabbit hearts [33] in response to a preconditioning stimulus.

Haemostatic proteins have also been implicated in ischemic preconditioning [34]. They activate fibrinolysis and reduce inflammation through mechanisms involving fibrinogen gamma [34]. In addition, fibrin beta decreases myocardial infarct size, scar formation, inflammation and the levels of cytokines (interleukin 1 beta, tumor necrosis factor-alpha and interleukin 6) in plasma [35]. Intravenous administration of fibrin-derived peptides is cardioprotective and reduces infarct size in rodents and pigs and appears to be as effective as preconditioning [35], [36]. Administration of fibrin beta to humans is reported to be safe with potential to protect against IR injury [36].

Transferrin was up-regulated during and after the RIPC. Although the exact involvement of transferrin in protection against IR injury is unknown, transferrin regulates production of reactive oxygen species via iron regulation and appears to have a protective role in IR injury [37], [38].

Although our analysis revealed proteins that are known to have a role during IR injury, there were also proteins whose role during IR injury is unknown. Our discussion is therefore centered on the proteins with known involvement in the IR injury. We were unable to obtain MS/MS data for all peptides that were changed significantly and these peptides require further analysis. The analysis involved matching peptide sequences against the sequence data of known proteins in the NCBInr human database. It is possible that there are proteins that have not been mapped in this database and therefore the origin of some of the detected peptides is not known. Peptides not identified in existing database searches may reflect programmed frame shifts or DNA sequencing errors [39].

A few studies suggested that a blood borne factor thought to be responsible for the ability of RIPC serum to transfer protection appears to have a molecular weight below 30 kDa [40]–[43]. Recently, Shimizu et al [40] demonstrated for the first time that transient limb ischemia liberates protective factors with molecular masses below 15 kDa, resistant to freezing and thawing, which is hydrophobic and not easily denatured. Serejo et al [42] have recently reported that the blood effluent from preconditioned rat hearts which was dialyzed to retain molecules above a molecular mass of 3500 Da had protective properties. On the other hand, Lang et al [43] reported that no differentially abundant proteins from RIPC with a known signalling function could be found above molecular mass of 8 kDa, the lower molecular mass limit of their proteomic study. Interestingly, Serejo et al [42] concluded that their finding “excludes the participation of adenosine (267.24 Da), opioids (500–800 Da), bradykinin (1060.22 Da), and other substances with molecular weights below the dialysis cutoff (3.5 kDa) as putative mediators of preconditioning”. These results still remain controversial and should be interpreted cautiously. At the present study, we found that some plasma proteins with molecular mass below 30 kDa coming from ischemic arm were up-regulated, for example, amiloidogenic transthyretin varients (15887 Da), apolipoprotein D (21276 Da), beta globin (2104 Da), haemoglobin alpha 2 (15258 Da), haemoglobin beta (15998 Da), vitamin D binding protein (2905 Da), while complement 8 gamma (22277 Da) was down-regulated. Proteomic assessment of the plasma taken from ischemic arms needs further scrutiny.

In the current study, the global proteomic responses to the RIPC stimulus reflected the genomic responses to the RIPC stimulus demonstrated in our previous study [9], in which there was a predominance of down-regulation of gene expression both early (at 15 minutes) and late (at 24 hours) after transient limb ischemia. We observed an increased number of down-regulated proteins in the early and even more so, during the late response to the RIPC stimulus.

Further research needs to be carried out to identify the pathways implicated in the RIPC response as well to identify the peptides and other metabolites that may be involved. This could be achieved by further depleting plasma of high abundance proteins to investigate those found in plasma at lower abundance. Furthermore, a proteomic assessment of plasma dialysate might be useful to assess the proteins with molecular weight of less than 15–30 kDa. If an effector of the RIPC stimulus is identified and its potency is properly enhanced, the application of such augmented RIPC could be immense, including all fields of cardiac surgery, organ transplantation, protection against stroke and post-cardiopulmonary bypass renal failure.

The study was designed to assess a global proteomic response to the RIPC and not to determine the proteins that may confer the protection. As such the study did not specifically assess the protein of low molecular weight.

### Conclusions

In summary, the results of this study demonstrate that the RIPC stimulus evokes a global proteomics response early and late, with predominant decrease in protein expression. There was an overall trend of up-regulation of protein expression in blood taken from the transiently ischemic limb during the RIPC protocol and this increase in the number of up-regulated peptides was cumulative with each cycle of the IR of the limb.
